# Physical and psychological changes of the COVID-19 infodemic by the older adult population

**DOI:** 10.1590/0034-7167-2023-0339

**Published:** 2024-09-06

**Authors:** Raísa Gonçalves Aquino, Eliane da Silva Pereira, Priscilla Alfradique de Souza, Graziele Ribeiro Bitencourt, Rosimere Ferreira Santana, Elaine Cristina Rodrigues da Costa, Ricardo Bezerra Cavalcante

**Affiliations:** I Universidade Federal do Estado do Rio de Janeiro. Rio de Janeiro, Rio de Janeiro, Brazil; II Universidade Federal Fluminense. Niterói, Rio de Janeiro, Brazil; III Universidade Federal do Rio de Janeiro. Macaé, Rio de Janeiro, Brazil; IV Universidade Federal de Juiz de Fora. Juiz de Fora, Minas Gerais, Brazil

**Keywords:** COVID-19, Aged, COVID-19 Pandemic, Information Dissemination, Mental Health, COVID 19, Adulto Mayor, Difusión de la Información, Pandemia del COVID-19, Salud Mental

## Abstract

**Objective:**

to analyze the physical and psychological changes of the COVID-19 infodemic for the older adult population of Rio de Janeiro.

**Method:**

a cross-sectional, web-based survey to find out about access to news and information about COVID-19 among older adult in Rio de Janeiro, between July and December 2020. Univariate analysis and bivariate analysis were carried out using non-parametric statistical methods.

**Results:**

390 older adults took part, predominantly female (75.1%), aged between 66 and 75 (35.4%), married (51.0%), white (60.3%), owning their own home (81.8%), located in urban areas (91%), with complete or incomplete primary education (31.8%) and retired or pensioners (79.2%). Younger people were significantly affected both physically and psychologically by social networks when compared to television (<0.001).

**Conclusion:**

Physical and psychological changes from exposure to information about COVID-19 have affected the lives of the older adult, having an impact on this population.

## RESUMEN

**Objetivo:**

Analizar las alteraciones físicas y psicológicas de la infodemia de COVID-19 en adultos mayores de Río de Janeiro.

**Método:**

Se trata de un estudio transversal, basado en la web, llevado a cabo para conocer el acceso a noticias e información sobre el COVID-19 entre adultos mayores de Río de Janeiro, realizado entre julio y diciembre de 2020. Se ejecutaron análisis univariados y bivariados utilizando métodos estadísticos no paramétricos

**Resultados:**

Participaron 390 adultos mayores, predominantemente mujeres (75,1%), con edades comprendidas entre los 66 y los 75 años (35,4%), casadas (51,0%), blancas (60,3%), vivienda propia (81,8%), situada en zona urbana (91%), estudios primarios completos o incompletos (31,8%) y jubiladas o pensionistas (79,2%). Los adultos más jóvenes fueron bastante afectados, tanto física como psicológicamente, por las redes sociales en comparación con la televisión (<0,001).

**Conclusiones:**

Los cambios físicos y psicológicos derivados de la exposición a la información sobre COVID-19 han afectado la vida de los adultos mayores de manera impactante.

## INTRODUCTION

With the emergence of the Coronavirus pandemic decreed in 2020, at the time of this pandemic emergency, there was a focus on the phenomenon of the “infodemic”, a term that means

a large increase in the volume of information associated with a specific subject, which can multiply exponentially in a short time due to a specific event, such as the COVID-19 pandemic. In this situation, rumors and misinformation arise, as well as the manipulation of information with dubious intent. In the information age, this phenomenon is amplified by social networks and spreads more quickly, like a virus^(1)^.

The volume of information that reaches people through various media can end up overwhelming them^(2)^. As a result, they often have issues involving anxiety, depression and exhaustion, which can make them unable to respond to the demands that arise^(1)^.

In this context, social media has enabled and brought ease and speed when it comes to disseminating news. Information is inherent to the period of development and maintenance of human societies. However, the media has expanded the production of this content and, due to the period of social distancing related to the pandemic, people are more exposed to the media, especially digital media, so that much of the news becomes accessible. However, verifying the veracity of this information is an important and difficult factor^(3)^.

In Brazil, the circulation of fake news is intense, especially their dissemination through social media - WhatsApp, Facebook and Instagram - and massive false information is spread, such as the non-existence of COVID-19 cases, with images of unoccupied hospital beds, and those that reported homemade methods to prevent contagion by the coronavirus, treatments without scientific proof of effectiveness, and conspiracy theories that attribute the pandemic to a political strategy and positions contrary to the social distancing measures necessary to contain the spread of the disease^(4, 5)^.

It is noteworthy that Rio de Janeiro is one of the states that according to data from the Brazilian Institute of Geography and Statistics (IBGE) is most populous in the country’s most aged age structure: Niterói, which is one of the municipalities in the state of Rio de Janeiro, has almost half a million inhabitants and has 96.8 thousand older adult (60 years old and over) for 80 thousand young people (0 to 14 years old), with a ratio of 120.9 older adult for every 100 young people^(6, 7)^.

The intersection of the COVID-19 pandemic with the infodemic is worrying, given that these media reach a large portion of the population. Among the age groups, the infodemic is a worrying phenomenon for the older adult population, considered the main risk group due to their high degree of vulnerability and susceptibility to complications and the need for hospitalization^(8, 9)^

In this way, the dissemination of intentionally false news has become a daily occurrence for the geriatric group. Sharing this information, even if it is not true, influences public opinion in general. This leads to treatments being abandoned and drug interactions, which can aggravate pre-existing illnesses and put the health of the older adult themselves and their families at risk^(10)^.

Based on this, it is relevant to invest in understanding how older people behave in the face of information about COVID-19 disseminated on the internet; what effects it has on their lives, and how anxiety, stress, fear and depression affect their mental health^(11)^.

## OBJECTIVES

To analyze the physical and psychological changes of the COVID-19 infodemic for the older adult population of Rio de Janeiro.

## METHODS

### Ethical aspects

The study was carried out in accordance with the ethical principles contained in Resolution 466/2012 and 512/2016 of the National Health Council, approved by the Research Ethics Committee of the Federal University of the State of Rio de Janeiro, complying with all the bioethical prerogatives of research with human beings. The digital Free and Informed Consent Form (FICF) was sent to the participants who agreed to take part in the research and were consequently directed to the questionnaire.

### Study design, period and location

This is a cross-sectional study, part of Phase 1 of a mixed multi-center study of an explanatory sequential strategy with a sample from the center of the state of Rio de Janeiro. The data collection period began on July 13, 2020 and lasted until December 30, 2020. The sample was calculated proportionally in all eight municipalities where the research was carried out. In each municipality, 20% was added for possible refusals in order to safeguard precision, considering the structure of the sampling plan, totaling 2,976 respondents for the 8 municipalities. The quality of the studies in the final sample was assessed using the Strengthening the Reporting of Observational Studies in Epidemiology (STROBE) checklist^(12)^. Respondents were invited to take part in the survey via social networks (Whatsapp, Facebook and Instagram) and/or e-mail and/or telephone, using the virtual snowball strategy by sending a link to the electronic questionnaire^(13)^.

### Population or sample; inclusion and exclusion criteria

The non-probabilistic sample had an estimated sample size of 390 older adult in the state of Rio de Janeiro. According to the IBGE census (2010) in the capital, the percentage of older adult is 14.64% of the total population of the municipality, reaching a total of 1,078,991 older adult. The sample size was calculated using the formula n = N.*Z* ^2^.*p*.(1 – *p*)/*Z* ^2^.p.(1 – *p*).+ *e* ^2^.(N – 1), where “n” is the calculated sample, “N” is the population, “Z” is the standardized normal variable associated with the confidence level, “p” is the true probability of the event and “(1-p)” the probability of the event not occurring (0.5 is the maximum variation assumption), finally “e” is the sampling error, using a 5% sampling error and a 95% confidence level.

The inclusion criteria were: people aged 60 or over, preserved cognitive functions, access to e-mail and/or social networks and/or telephone. Exclusion criteria were not being able to answer the questionnaire using digital media or even by telephone.

### Study protocol

Participants were invited to take part in the survey using the snowball method, which is defined as a sampling strategy that uses chains of reference, such as a network, to collect information using chain recruitment techniques^(13)^. As this was a virtual snowball sample, there was an increase of 10.01% in the final sample under study, going beyond the municipalities where the study was based.

Data was collected using a web-based survey (via e-mail and/or social networks and/or telephone), due to the difficulty in accessing older adult in social isolation. The web-based survey was sent (three attempts over three months) to older adult (aged 60 or over) with access to e-mail and/or social networks. The link to access the web-based survey was sent to scientific societies of geriatrics and gerontology, care institutions for the older adult, pensioners’ associations and directly to possible older adult already being monitored by the centers taking part in the research. Pilot interviews were carried out to adapt the questionnaire, minimizing potential sources of bias.

The study variables included demographic and socioeconomic characteristics: gender (categorized as male, female and undeclared), age group (60-69 years, 70-79 years, 80 years or more), marital status (with partner, without partner), race/color (white, black, brown, other), number of people living in the house (lives alone, with one to two people, with three or more people), condition of residence (own, other), location of residence (urban area, rural area), education level (primary, secondary, higher and higher), use of health services (Unified Health System - SUS, private, both), number of dependents (no dependents, one to two, three or more), source of income (none, one source of income, more than 1 source of income), change in income with the COVID-19 pandemic (income did not change or increased, income decreased).

Variables relating to exposure to news and information about COVID-19: frequency of exposure per day on social networks, television and radio, exposure in the last week (exposed, not exposed) in the different media (social networks, television, radio), information vehicles most used to access news and information (social networks, television, radio, newspapers or printed magazines, none, other), information from social networks, television or radio has affected (analyzed dichotomized into yes and no and also categorized into does not use this medium, does not affect, affects physically, affects psychologically, physically and psychologically), feelings of fear, awareness, stress, security, anxiety that this information generated (some response, no response) when it referred to the number of people infected and killed by COVID-19, fear related to the disease, photos, videos and fake news about COVID-19, broadcast on social media, television and radio, frequency of signs and symptoms observed when exposed to information about COVID-19 in the last 15 days, assessed by the sum of points per category (never, a few times, sometimes, often) and dichotomized based on positive screening for psychological distress through exposure to information about COVID-19 (case and non-case).

The Perceived Stress Scale (PSS) was also used, consisting of 14 questions related to the experiences they have had in the past month, in terms of whether they are unpredictable, uncontrollable and how much they burden their lives^(14)^. The Likert-type scale uses the frequency (never, almost never, sometimes, almost always, always) with which the feelings and thoughts were perceived, ranging from 0 to 4 points. The items are divided into seven negative and seven positive, and in the case of the positive items the score is decreasing for the overall calculation. The final score can vary from 0 to 56 points, and the higher the score, the greater the level of stress perceived by the individual. It is understood that the results should be analyzed based on the average score, since grouping scores of continuous variables leads to a loss of sensitivity^(15, 16)^.

The Geriatric Depression Scale (GDS)^(17)^, the 15-item version of the questionnaire, built with 30 questions to be answered on the basis of how the older adult person felt the previous week, was validated for Brazil in a reduced version of 15 items, 10 of which receive a score if answered positively and the other 5 items receive a score if answered negatively. This 15-item version was used in this study. It has a final score from 0 to 15, where zero represents the absence of depressive symptoms and fifteen indicates the greatest burden of these symptoms. The cut-off points used are as follows: 0 to 5 (normal), 6 to 10 (mild depressive symptoms) and 11 to 15 (severe depressive symptoms)^(18)^.

To measure anxiety in the older adult population, a widely used scale is the Geriatric Anxiety Inventory (GAI). Initially developed in an Australian study, the GAI determined, for its target population, 8/9 as the cut-off point to indicate the presence of an anxiety disorder^(19)^. The Geriatric Anxiety Inventory (GAI-BR) showed an average score of 8.77 and an inverse correlation with family income and educational level. The cut-off point of 13 indicated that the GAI-BR was able to discriminate individuals with Generalized Anxiety Disorder (GAD) from those without GAD. For the screening of anxious symptoms, 8 is suggested as the cut-off point, although the low specificity found undermines the overall performance of the scale^(18)^.

### Analysis of results and statistics

The data collected through the electronic form was entered and stored in a Microsoft Office Excel® 2010 spreadsheet, according to the coding determined for each of the variables of interest in the study, feeding into a general database including all the municipalities.

The data was analyzed using R 4.1.1. A univariate analysis was carried out to describe the profile of the study participants. This was followed by a bivariate analysis using non-parametric statistical methods. The Mann-Whitney test^(20)^, Kruskal Wallis test^(20)^, Dunn’s multiple comparison test, Chi-square test and Fisher’s exact test^(21)^ were used. The Mann-Whitney test and the Kruskal-Wallis test were used for independent samples. Dunn’s multiple comparisons test was applied after showing significant differences in at least one of the response categories using the Kruskal-Wallis test.

The estimates were presented considering a significance level of 5% (p < 0.05) in all analytical procedures. The confidence interval adopted was 95%. The results were described and presented in tables.

## RESULTS

The final sample was characterized predominantly by women (75.1%), aged between 66 and 75 (35.4%), married (51.0%), white (60.3%), living in their own home (81.8%) located in the urban area (91%), with complete or incomplete primary education (31.8%), having a pension or pensioner as a source of income (79.2%) and with one to two dependents on their income (61%) (Table 1).

**Table 1:** Sociodemographic characteristics compared to access to social media. Rio de Janeiro, Rio de Janeiro, Brazil, 2022

Variables	Television			*p* value^2^	Social network		*p* value^2^	Radio		*p* value^2^
	Total n(%)	No access N = 58^1^	WITH access N = 332^1^		No access N = 231^1^	WITH access N = 159^1^		No access N = 299^1^	WITH access N = 91^1^	
Sex				0.889			0.044*			0.004*
Feminine	293 (75.1)	44 (75.9)	249 (75.0)		182 (78.8)	111 (69.8)		235 (78.6)	58 (63.7)	
Masculine	97 (24.9)	14 (24.1)	83 (25.0)		49 (21.2)	48 (30.2)		64 (21.4)	33 (36.3)	
Age				<0.001*			<0.001*			0.088
60 – 65	134 (34.4)	32 (55.2)	102 (30.7)		73 (31.6)	61 (38.4)		107 (35.8)	27 (29.7)	
66 – 75	138 (35.4)	18 (31.0)	120 (36.1)		67 (29.0)	71 (44.7)		97 (32.4)	41 (45.1)	
≥76	118 (30.3)	8 (13.8)	110 (33.1)		91 (39.4)	27 (17.0)		95 (31.8)	23 (25.3)	
Marital status				0.093			0.017*			0.070
Married	199 (51.0)	23 (39.7)	176 (53.0)		104 (45.0)	95 (59.7)		151 (50.5)	48 (52.7)	
Widower	73 (18.7)	10 (17.2)	63 (19.0)		53 (22.9)	20 (12.6)		54 (18.1)	19 (20.9)	
Separated	67 (17.2)	16 (27.6)	51 (15.4)		43 (18.6)	24 (15.1)		59 (19.7)	8 (8.8)	
Single	51 (13.1)	9 (15.5)	42 (12.7)		31 (13.4)	20 (12.6)		35 (11.7)	16 (17.6)	
Cohabitants				0.176			0.299			0.192
None	71 (18.2)	15 (25.9)	56 (16.9)		46 (19.9)	25 (15.7)		55 (18.4)	16 (17.6)	
1 person	145 (37.2)	15 (25.9)	130 (39.2)		89 (38.5)	56 (35.2)		119 (39.8)	26 (28.6)	
2 people	93 (23.8)	16 (27.6)	77 (23.2)		55 (23.8)	38 (23.9)		66 (22.1)	27 (29.7)	
≥3 people	81 (20.8)	12 (20.7)	69 (20.8)		41 (17.7)	40 (25.2)		59 (19.7)	22 (24.2)	
Property type				0.009			0.478			0.353
Own	319 (81.8)	40 (69.0)	279 (84.0)		185 (80.1)	134 (84.3)		241 (80.6)	78 (85.7)	
Leased	46 (11.8)	14 (24.1)	32 (9.6)		31 (13.4)	15 (9.4)		36 (12.0)	10 (11.0)	
of family	25 (6.4)	4 (6.9)	21 (6.3)		15 (6.5)	10 (6.3)		22 (7.4)	3 (3.3)	
Education level				<0.001|*			<0.001			0.004*
Illiterate	9 (2.3)	0 (0.0)	9 (2.7)		8 (3.5)	1 (0.6)		4 (1.3)	5 (5.5)	
Complete or incomplete	124 (31.8)	11 (19.0)	113 (34.0)		98 (42.4)	26 (16.4)		84 (28.1)	40 (44.0)	
basic education										
High school	108 (27.7)	8 (13.8)	100 (30.1)		59 (25.5)	49 (30.8)		86 (28.8)	22 (24.2)	
Graduation	70 (17.9)	14 (24.1)	56 (16.9)		27 (11.7)	43 (27.0)		58 (19.4)	12 (13.2)	
Postgraduate	79 (20.3)	25 (43.1)	54 (16.3)		39 (16.9)	40 (25.2)		67 (22.4)	12 (13.2)	

^1^ *n (%);* ^2^ Pearson’s Chi-squared test; Fisher’s exact test; *p value < 0,05; sd = standard deviation.

In terms of analysis, the older adult said they had been exposed to news or information about COVID-19 frequently over the last week, 38.5% of which was on television, 22.3% on social media and 8.7% on the radio. The following were most frequently cited as being used to access news and information about COVID-19: television (85.1%), Whatsapp (40.8%), internet sites (28.7%), newspapers or printed magazines (26.2%), Facebook (25.1%), radio (23.3%), Youtube (14.4%), Instagram (7.7%), Twitter (3.3%), Telegram (1.8%), none of the above (1. 5%), Scientific papers forwarded (0.5%), Scientific articles (0.3%), Google (0.3%) and Emergency hospital (0.3%).

As for the sociodemographic variables related to access to news and information about COVID-19, in the comparison of exposure between the different social media (social networks and television), the variables were similar (Table 2). The exception was the variables age and education, which were significant (p value <0.001). In the exposure groups, where it has affected them physically and/or psychologically, the main groups were young older adult with complete elementary and high school education.

**Table 2:** Sociodemographic characteristics related to access to news and information about COVID-19. Rio de Janeiro, Rio de Janeiro, Brazil, 2022

Sociodemographic profile	Social media				*p* value^2^	Television			*p* value^2^
	Total N = 390^1^	I do not “use” this mean of information N = 163^1^	It hasn’t affected me N = 114^1^	It has affected me physically and/or psychologically N = 113^1^		I do not “use” this mean of information N = 163^1^	It hasn’t affected me N = 114^1^	It has affected me physically and/or psychologically N = 113^1^		*p* value^2^
Sex					0.347				0.640
Female	293 (75.1)	126 (77.3)	80 (70.2)	87 (77.0)		22 (73.3)	131 (73.)	140 (77.3)	
Male	97 (24.9)	37 (22.7)	34 (29.8)	26 (23.0)		8 (26.7)	48 (26.8)	41 (22.7)	
Age					<0.001				<0.001
60 - 65 years old	134 (34.4)	31 (19.0)	40 (35.1)	63 (55.8)		12 (40.0)	42 (23.5)	80 (44.2)	
66 - 75 years old	138 (35.4)	49 (30.1)	52 (45.6)	37 (32.7)		11 (36.7)	64 (35.8)	63 (34.8)	
76 years or older	118 (30.3)	83 (50.9)	22 (19.3)	13 (11.5)		7 (23.3)	73 (40.8)	38 (21.0)	
Marital status					0.071				0.578
Married/living	199 (51.0)	80 (49.1)	59 (51.8)	60 (53.1)		15 (50.0)	93 (52.0)	91 (50.3)	
together									
Widower	73 (18.7)	42 (25.8)	13 (11.4)	18 (15.9)		4 (13.3)	35 (19.6)	34 (18.8)	
Separated/separated	67 (17.2)	22 (13.5)	24 (21.1)	21 (18.6)		4 (13.3)	27 (15.1)	36 (19.9)	
Single	51 (13.1)	19 (11.7)	18 (15.8)	14 (12.4)		7 (23.3)	24 (13.4)	20 (11.0)	
Cohabitants					0.177				0.280
None	71 (18.2)	29 (17.8)	19 (16.7)	23 (20.4)		5 (16.7)	30 (16.8)	36 (19.9)	
1 person	145 (37.2)	63 (38.7)	43 (37.7)	39 (34.5)		10 (33.3)	73 (40.8)	62 (34.3)	
2 people	93 (23.8)	41 (25.2)	33 (28.9)	19 (16.8)		10 (33.3)	46 (25.7)	37 (20.4)	
3 people or more	81 (20.8)	30 (18.4)	19 (16.7)	32 (28.3)		5 (16.7)	30 (16.8)	46 (25.4)	
Type of property					0.220				0.135
Own residence	319 (81.8)	134 (82.2)	95 (83.3)	90 (79.6)		24 (80.0)	145 (81.0)	150 (82.9)	
Rented residence	46 (11.8)	17 (10.4)	10 (8.8)	19 (16.8)		3 (10.0)	18 (10.1)	25 (13.8)	
Family residence	25 (6.4)	12 (7.4)	9 (7.9)	4 (3.5)		3 (10.0)	16 (8.9)	6 (3.3)	
Education level					<0.001				<0.001
Illiterate	9 (2.3)	7 (4.3)	1 (0.9)	1 (0.9)		0 (0.0)	6 (3.4)	3 (1.7)	
Complete or incomplete	124 (31.8)	79 (48.5)	23 (20.2)	22 (19.5)		0 (0.0)	66 (36.9)	58 (32.0)	
basic education									
High school	108 (27.7)	46 (28.2)	27 (23.7)	35 (31.0)		9 (30.0)	52 (29.1)	47 (26.0)	
Graduation	70 (17.9)	17 (10.4)	32 (28.1)	21 (18.6)		11 (36.7)	32 (17.9)	27 (14.9)	
Postgraduate	79 (20.3)	14 (8.6)	31 (27.2)	34 (30.1)		10 (33.3)	23 (12.8)	46 (25.4)	

^1^ *n (%);* ^2^ Pearson’s Chi-squared test; Fisher’s exact test; *p value < 0,05; sd = standard deviation.

In terms of hours of exposure to news and information about COVID-19, the average number of hours of exposure was: 5.3 hours for television, 6.8 hours for social networks (Whatsapp, Facebook, Youtube, Instagram and others) and 4.7 hours for radio, as scored in Table 3. There was significance (p <0.001) in relation to the time of exposure and the Social Network, and it should be noted that this significance was obtained in the group of older adult (n=111) who said they were affected by the news and information about COVID-19 broadcast on the Social Network. An important piece of data to bring to light in the scientific discussion was that the largest number of older adult (n=177) affected by exposure to news about COVID-19 was through the television.

**Table 3:** Hours of exposure to news and information about COVID-19 - exposure time does or does not affect the older adult. Rio de Janeiro, Rio de Janeiro, Brazil, 2022

Exposure hours	Affects	Psychologically	Physically	Both	*p* value^2^
Social network	n = 111	n = 75	n = 12	n= 24	<0.001*
Mean (sd)	6.8 (6.1)	7.4 (6.2)	4.4 (4.4)	6.2 (6.4)	
Median (q1;q3)	4.0 (2.0 – 10.0)	4.0 (3.0 – 12.0)	2.0 (1.0 – 6.2)	3.5 (2.0 – 8.0)	
Television	n = 177	n = 120	n = 25	n = 32	0.203
(sd)	5.3 (4.8)	5.1 (4.6)	4.5 (3.3)	6.6 (6.4)	
Median (q1;q/3)	4.0 (2.0 – 7.0)	4.0 (2.0 – 6.2)	4.0 (2.0 – 5.0)	4.5 (2.0 – 8.7)	
Radio	n = 46	n = 33	n = 6	n = 7	0.008
Mean (sd)	4.7 (5.7)	3.7 (4.3)	5.0 (2.0)	9.5 (10.5)	
Median (q1;q3)	3.0 (2.0 – 4.7)	2.0 (1.0 – 4.0)	5.0 (4.0 – 6.0)	3.0 (1.5 – 18.0)	

^1^ *n (%);* ^2^ Pearson’s Chi-squared test; Fisher’s exact test; * p value < 0,05; sd = standard deviation.

## DISCUSSION

At the same time as there is a perception that these older adult have access to content via the internet, there is a need for greater understanding of the real comprehension of the information received, especially during the pandemic period in which the study was developed, since it is from this notion and understanding that the impact of the various information can become an influencing factor in the lives of the older adult population in particular.

Other web-based survey studies conducted during the pandemic in Brazil also reflect a greater number of older adult women, but with an age range of 60 to 69 years, married, with more schooling and living with other people. An analysis of the sociodemographic profile of the sample in this study reveals a new pattern among older adult who use digital media, older adult who are educated to a level of comprehension and reading that allows them to access knowledge via the internet, corroborating other studies^(22)^.

In terms of exposure to news or information about Covid-19 obtained by the older adult, television presented the highest percentage. Thus, younger older adult with a higher level of education have more exposure to television and social media. Data from the IBGE shows that the older adult have a monthly household income per capita of up to half the minimum wage, with only 10.8% having electricity, a computer, color television and a washing machine at the same time^(6, 7, 8, 9, 10, 11, 12, 13, 14, 15, 16, 17, 18, 19, 20, 21, 22, 23)^.

According to surveys, the majority of people’s leisure time is spent watching television, with 93% watching television at home and 72% saying that they prefer activities carried out at home^(24)^. A study among older adult Brazilians reaffirmed the use of television over other media. It also raised concerns about the impact of too much exposure on the physical health, psychological condition and quality of life of these users^(25)^. It is noteworthy that television was the main source of information among older adult living in the Southeast and Midwest regions and the Internet among residents of the North, Northeast and South, as well as the findings of this study^(26)^.

With regard to the significance of the associations established between social media and sociodemographic variables, age was significant in two social media: television and social networks, and schooling was significant in television and radio. Analyzing these cross-significances with sociodemographic variables are factors that influence how the older adult understand the information they receive and, consequently, how this information can impact the quality of their physical and mental health.

When sociodemographic variables were associated as a factor in whether the older adult felt affected psychologically, physically or both due to the news broadcast on social media, linked to all the exposure to information about COVID-19, it was observed that the greatest burden of these changes was found in older adult with higher levels of education and lower income in the post-pandemic period^(27)^.

Most of the older adult reported having higher education or a higher level of education, something that raises questions and critical thinking when promoting the articulation of how much the older adult can be affected psychologically, physically or both in which the longer the study period, the more impacts can be evidenced in the face of too much information and without scientific screening conveyed by the various media.

In a scoping review, it was possible to identify an association between a higher level of education and the chance of developing anxiety and depression. In this way, it was inferred that the level of education could be related both to understanding the seriousness of the pandemic situation and to seeking information about COVID-19^(28)^. However, another study also conducted with the older adult population in the southeast region of Brazil found that more educated older adult had lower rates of depression and loneliness due to greater options for resilience strategies and social support^(22)^.

There is a progressive increase in internet use among older adult Brazilians, reaching 97% by 2021^(29)^. Access to social networks presents a duality, because if on the one hand access to them can lead to greater autonomy for the older adult, availability of information and fostering interpersonal relationships, on the other hand it presents challenges such as infodemia, the difficulty of controlling the information conveyed, thus making the older adult not know which orientation to follow, which can have an impact on their physical and/or psychological health^(30)^.

The psychological consequences of excessive use of social media, especially during the COVID-19 pandemic, have led to increasingly complex changes. Among these, the psychological disorders most reported in the studies were divided into four variables encompassing: anxiety, worry and fear; stress; depression and suicidal thoughts^(31)^. An Iraqi study of 516 users regarding the impact of panic on social media during the pandemic showed a significant impact on mental health and well-being, especially in terms of increased levels of fear, stress, depression and suicidal thoughts^(32)^.

### Study limitations

A limiting issue in the study was the data collection strategy using a web-based survey, because even though many older adult have access to the internet, there are still barriers that make it impossible for them to be able to effectively answer a survey online. The study used a non-random sample, so caution is needed when interpreting causal relationships between variables and generalizing the findings of this study to the older adult population.

### Contributions to the field of Nursing

It is extremely important to investigate issues related to social media and how they can affect the health of the older adult, especially during periods of pandemic such as the COVID-19 pandemic. As a contribution, the study highlights the constant need to discuss scientific evidence regarding issues that alter the mental and physical health of the older adult population, in order to contribute to public policies aimed at promoting and maintaining the psychological and physical well-being of the older adult. In addition, to propose new studies with strategies to minimize the negative impacts that may arise from the COVID-19 infodemic.

## CONCLUSION

Physical and psychological changes do exist and are correlated with exposure to information about COVID-19 in the lives of the older adult, which has made it possible to identify the impact suffered by this population. Although it is a contemporary issue, there is still a need for greater multi-professional exploration, especially by gerontological nursing, given that any type of physical and psychological alteration will interfere with the health profile of the older adult. Understanding these repercussions is necessary in order to propose effective individual and collective interventions to deal with infodemia in this population group.

It is essential that there are always scientific discussions about the mental health and well-being of the population, especially at times as specific as the COVID-19 pandemic. Thus, the study made it possible to visualize the physical and psychological changes of the COVID-19 infodemic for the older adult population of Rio de Janeiro, and it was also possible to validate the significance of social media for the older adult and how much the news broadcasts affect this population.
