# Supplementary material for: Physical and psychological changes of the COVID-19 infodemic by the older adult population
Source: Rev Bras Enferm. 2024 Sep 6;77(4):e20230339. doi: 10.1590/0034-7167-2023-0339 (PMC11382676; doi:10.1590/0034-7167-2023-0339)
Supplement: Translation

# Translation: Alterações físicas e psicológicas da infodemia de COVID-19 pela população idosa

**DOI:** 10.1590/0034-7167-2023-0339pt

**Authors:** Raísa Gonçalves Aquino, Eliane da Silva Pereira, Priscilla Alfradique de Souza, Graziele Ribeiro Bitencourt, Rosimere Ferreira Santana, Elaine Cristina Rodrigues da Costa, Ricardo Bezerra Cavalcante

**Affiliations:** I Universidade Federal do Estado do Rio de Janeiro. Rio de Janeiro, Rio de Janeiro, Brasil; II Universidade Federal Fluminense. Niterói, Rio de Janeiro, Brasil; III Universidade Federal do Rio de Janeiro. Macaé, Rio de Janeiro, Brasil; IV Universidade Federal de Juiz de Fora. Juiz de Fora, Minas Gerais, Brasil

**Keywords:** COVID-19, Idoso, Disseminação de Informação, Pandemia, Saúde Mental

## Abstract

**Objetivo:**

Analisar as alterações físicas e psicológicas da infodemia de COVID-19 para a população idosa do Rio de Janeiro.

**Método:**

estudo transversal, *web-based survey.* para conhecimento do acesso a notícias e informações sobre COVID-19 de idosos do Rio de Janeiro, durante o período de julho a dezembro de 2020. Realizou-se análise univariada e análise bivariada com aplicação de métodos estatísticos não paramétricos.

**Resultados:**

Participaram 390 idosos, predominantemente do sexo feminino (75,1%), faixa etária entre 66 e 75 anos (35,4%), casados (51,0%), raça/cor branca (60,3%), com imóvel próprio (81,8%), localizado na zona urbana (91%), ensino básico completo ou incompleto (31,8%) e aposentados ou pensionistas (79,2%). Idosos mais jovens foram significativamente afetados tanto física quanto psicologicamente pelas redes sociais quando comparado a televisão (<0,001).

**Conclusão:**

Alterações físicas e psicológicas da exposição às informações sobre a COVID-19 têm afetado a vida dos idosos, gerando impacto para esta população.

## INTRODUÇÃO

Com o surgimento da pandemia do Coronavirus (COVID-19), decretada em 2020, houve um enfoque no fenômeno da “infodemia”, termo este que significa

um grande aumento no volume de informações associadas a um assunto especifico, que pode se multiplicar exponencialmente em pouco tempo devido a um evento especifico, como a pandemia de COVID-19. Nessa situação, surgem rumores e desinformação, além da manipulação de informações com intenção duvidosa. Na era da informação, o fenômeno é amplificado pelas redes sociais e se alastra mais rapidamente, como um vírus^(1)^.

O volume de dados que alcança as pessoas por diversas mídias pode acabar por sobrecarregá-las^(2)^. Consequentemente, muitas vezes, desperta-se questões que envolvem ansiedade, depressão, exaustão, o que pode fazer com que tenham incapacidade de responder às demandas que se apresentam^(1)^.

Dessa forma, as mídias sociais viabilizaram a facilidade e a velocidade quando se trata de disseminação de noticias. As informações são inerentes ao período de desenvolvimento e manutenção das sociedades humanas. Entretanto, os meios de comunicação ampliaram a produção de conteúdos e, devido ao período de distanciamento social relacionado à pandemia, as pessoas estão mais expostas às mídias, enfatizando as digitais, de modo que boa parte das notícias se torna acessível. Porém, a verificação da veracidade dessas informações é um fator importante e dificultado^(3)^.

No Brasil, a circulação de notícias falsas é intensa, destaca-se a veiculação delas por meio das mídias sociais – *WhatsApp, Facebook eInstagram –* a exemplo de divulgações sobre a inexistência de casos de COVID-19, com imagens de leitos hospitalares desocupados, bem como os relatos de métodos caseiros para a prevenção do contágio do coronavírus, tratamentos sem comprovação científica de eficácia, e teorias conspiratórias que atribuem a pandemia a uma estratégia política e posicionamentos contrários às medidas de distanciamento social necessárias à contenção da propagação da doença^(4, 5)^.

Destaca-se que o Rio de Janeiro é um dos estados, conforme os dados do Instituto Brasileiro de Geografia e Estatística (IBGE), mais populosos na estrutura etária mais envelhecida do país: Niterói, que é um dos municípios do estado do Rio de Janeiro, possui quase meio milhão de habitantes e obtinha 96,8 mil idosos (60 anos e mais) para 80 mil jovens (0 a 14 anos), com uma relação de 120,9 idosos para cada 100 jovens^(6, 7)^.

O cruzamento da pandemia de COVID-19 com a infodemia torna-se algo preocupante, sobretudo tendo em vista que essas mídias alcançam elevada parcela da população. Dentre os grupos etários, a infodemia é um fenômeno preocupante para os idosos, já que estes constituem o principal grupo de risco diante do elevado grau de vulnerabilidade à desinformação e a suscetibilidade para complicações e necessidade de internação^(8, 9)^.

Dessa maneira, a divulgação de notícias intencionalmente falsas se tornou algo cotidiano para o grupo geriátrico à medida que o seu compartilhamento influencia a opinião pública em geral. Isso propicia o abandono de tratamentos, suscita interações medicamentosas podendo, assim, agravar doenças preexistentes e colocando em risco a saúde dos próprios idosos e de seus familiares^(10)^.

A partir disso, é relevante investir na compreensão de como as pessoas idosas comportam-se frente às informações sobre a COVID-19 disseminadas pela internet; que efeitos provocam em suas vidas, e como a ansiedade, o estresse, o medo e a depressão repercutem em sua saúde mental^(11)^.

## OBJETIVO

Analisar as alterações físicas e psicológicas da infodemia de COVID-19 para a população idosa do Rio de Janeiro.

## MÉTODOS

### Aspectos éticos

O estudo foi realizado conforme os princípios éticos que constam na Resolução 466/2012 e 512/2016 do Conselho Nacional de Saúde, aprovado pelo Comitê de Ética em Pesquisa da Universidade Federal do Estado do Rio de Janeiro, cumprindo-se todas as prerrogativas bioéticas da pesquisa com seres humanos. Encaminhou-se o Termo de Consentimento Livre e Esclarecido (TCLE) digital para aqueles que aceitaram participar da pesquisa e, consequentemente, foram direcionados ao questionário.

### Desenho, período e local do estudo

Trata-se de um estudo transversal, em que se parte da Fase 1 de um estudo multicêntrico misto de estratégia sequencial ex-planatória com a amostra do centro do estado do Rio de Janeiro. O período de coleta de dados iniciou-se em 13 de julho de 2020 e perdurou até 30 de dezembro de 2020. A amostra foi calculada proporcionalmente em todos os oito municípios-sede de pesquisa. Em cada município, adicionou-se 20% para eventuais recusas de modo a resguardar precisão, considerando a estrutura do plano amostral, totalizando 2.976 respondentes para os 8 municípios. A qualidade dos estudos da amostra final foi avaliada por meio da lista de verificação, *Strengthening the Reporting of Observational Studies in Epidemiology (STROBE)* ^(12)^. Os respondentes, por envio de link do questionário eletrônico, foram convidados a participar da pesquisa via redes sociais (*Whatsapp, Facebooke Instagram*) e/ou e-mail e/ou telefone, utilizando-se a estratégia bola de neve virtual^(13)^.

### População ou amostra; critérios de inclusão e exclusão

A amostragem não probabilística contou com um tamanho amostral estimado de 390 idosos no Estado do Rio de Janeiro. De acordo com o censo do IBGE (2010), na capital, a porcentagem de pessoas idosas é de 14,64% da população total do município, chegando à soma de 1.078.991 mil idosos. O cálculo do tamanho da amostra foi estimado utilizando-se a fórmula n = N.*Z* ^2^.p.(1 – *p*)/*Z* ^2^. *p*.(1 – *p*).+ *e*2.(N – 1), em que “n” é a amostra calculada, “N” é a população, “Z” é a variável normal padronizada associada ao nível de confiança, “p” é a verdadeira probabilidade do evento e “(1-p)” a probabilidade do evento não ocorrer (0,5 é a suposição de variação máxima). Finalmente, “e” é o erro amostral, sendo utilizados erro amostrai de 5% e um nível de confiança de 95%.

Os critérios de inclusão foram: pessoas com 60 anos ou mais, funções cognitivas preservadas, acesso a e-mail e/ou redes sociais e/ou telefone. Já para exclusão: declarar não possuir habilidade para responder o questionário utilizando as mídias digitais ou mesmo pelo telefone.

### Protocolo do estudo

A captação ou o convite para os participantes na pesquisa ocorreu pelo método bola de neve, define-se como uma estratégia de amostragem que utiliza de cadeias de referência, como uma rede para coleta de informações usando técnicas de recrutamento em cadeia^(13)^. Por se tratar de amostragem pelo método bola de neve virtual, houve um acréscimo na amostra final em estudo de 10,01% extrapolando municípios-sede.

A coleta de dados foi realizada pela *web-based survey* (por e-mail e/ou redes sociais e/ou telefone), pela dificuldade em acessar os idosos em isolamento social. A *web-based survey* foi enviada (três tentativas por três meses) para idosos (60 anos ou mais) com acesso a *e-mail* e/ou redes sociais. O link para acesso à *web-based survey* foi encaminhado para sociedades científicas de geriatria e gerontologia, instituições de assistência a idosos, associações de aposentados e diretamente a possíveis idosos já acompanhados pelos centros participantes da pesquisa. Foram realizadas entrevistas-piloto para adequação do questionário, minimizando potenciais fontes de viés.

As variáveis do estudo contaram com características demográficas e socioeconômicas: sexo (categorizado em masculino, feminino e não declarado); faixa etária (60-69 anos, 70-79 anos, 80 anos ou mais); situação conjugal (com companheiro, sem companheiro); raça/cor (branca, preta, parda, outras); número de pessoas residentes na casa (mora sozinho, com uma a duas pessoas, com três ou mais pessoas); condição da residência (própria, outra); localização da residência (área urbana, área rural); escolaridade (fundamental, médio, superior e maior); utilização dos serviços de saúde (Sistema Único de Saúde - SUS, privado, ambos); número de dependentes da renda (nenhum dependente, uma a duas, três ou mais); fonte de renda (nenhuma, uma fonte de renda, mais de 1 fonte de renda); alteração da renda com a pandemia de COVID-19 (renda não alterou ou aumentou, renda diminuiu).

As variáveis referentes à exposição às notícias e informações sobre COVID-19 são: frequência de exposição por dia nas redes sociais, na televisão e no rádio; exposição na última semana (se foi exposto ou não exposto) nas diferentes mídias (redes sociais, televisão, rádio); veículos de informação mais utilizados para acessar notícias e informações (redes sociais, televisão, rádio, jornais ou revistas impressas, nenhuma, outro); se informações das redes sociais, televisão ou rádio tem afetado (de modo que a análise foi dicotomizada em sim e não e também categorizada em: não utiliza este meio, não afeta, afeta fisicamente, afeta psicologicamente, física e psicologicamente), considerando sentimentos de medo, conscientização, estresse, segurança, ansiedade que essas informações geravam (alguma resposta, nenhuma resposta) quando faziam referência ao número de infectados e mortos por COVID-19. Ainda em relação às variáveis, verificou-se acerca do medo relacionado à doença em fotos, vídeos e notícias falsas sobre COVID-19, veiculadas nas redes sociais, televisão e rádio, além da frequência de sinais e sintomas observados quando foram expostos às informações sobre COVID-19 nos últimos 15 dias, sendo avaliada pelo somatório de pontos por categoria (nunca, poucas vezes, algumas vezes, muitas vezes) e dicotomizada a partir do rastreio positivo para sofrimento psíquico mediante a exposição a informações sobre COVID-19 (caso e não caso).

Utilizou-se, ainda, a Escala de Estresse Percebido (EEP) composta por 14 questões relacionadas às experiências vivenciadas, no período passado de um mês, quanto ao fato de serem imprevisíveis, incontroláveis e em qual intensidade sobrecarregam sua vida^(14)^. A escala do tipo *Likert* utiliza a frequência (nunca, quase nunca, às vezes, quase sempre, sempre) com que os sentimentos e pensamentos foram percebidos, variando de 0 a 4 pontos. Os itens são divididos em sete negativos e sete positivos, sendo que, no caso dos itens positivos, a pontuação é decrescente para o cômputo geral. A pontuação final pode variar de 0 a 56 pontos e quanto mais alto o escore, maior o nível de estresse percebido pelo indivíduo. Entende-se que a análise dos resultados deve ser feita a partir do escore médio, uma vez que agrupar escores de variáveis contínuas acaba levando à perda de sensibilidade^(15, 16)^.

A Escala de Depressão em Geriatria (EDG)^(17)^, construída com 30 questões que deveriam ser respondidas a partir do que o idoso sentiu na semana anterior, foi validada para o Brasil na versão reduzida de 15 itens, em que 10 deles recebem pontuação se respondidos positivamente e os outros 5 itens pontuam caso respondidos negativamente. A versão com 15 itens foi a utilizada neste estudo. Ela possui pontuação final de 0 a 15, em que zero representa a ausência de sintomas depressivos e quinze indica a maior carga desses sintomas. Os pontos de corte utilizados são os seguintes: 0 a 5 (normal), 6 a 10 (sintomas depressivos leves) e 11 a 15 (sintomas depressivos graves)^(18)^.

Para aferir a ansiedade na população idosa, uma escala amplamente utilizada é o *Geriatric Anxiety Inventory* (GAI). Desenvolvido inicialmente em um estudo australiano, o GAI determinou, para sua população-alvo, os valores 8/9 como ponto de corte para indicar a presença de transtorno de ansiedade^(19)^. Inventário de Ansiedade Geriátrica (GAI-BR), apresentou escore médio de 8,77 e correlação inversa com renda familiar e nível educacional. O ponto de corte 13 indicou que o GAI-BR foi capaz de discriminar indivíduos com Transtorno de Ansiedade Generalizada (TAG) daqueles sem TAG. Já para o rastreio de sintomas ansiosos, sugere-se 8 como ponto de corte, embora a baixa especificidade encontrada prejudique o desempenho geral da escala^(18)^.

### Análise dos resultados e estatística

Os dados coletados através do formulário eletrônico foram digitados e armazenados em uma planilha eletrônica no programa *Microsoft Office Excel*® 2010, conforme codificação determinada para cada uma das variáveis de interesse do estudo, alimentando um banco de dados geral, incluindo todos os municípios.

O estudo dos dados foi realizado no R 4.1.1. Desse modo, fez-se uma análise univariada com a finalidade de descrever o perfil dos participantes da pesquisa. Em seguida, fez-se a análise bivariada com aplicação de métodos estatísticos não paramétricos. Assim, utilizou-se o teste de Mann-Whitney^(20)^, teste de Kruskal Wallis^(20)^, o teste de comparações múltiplas de Dunn, teste de Qui-quadrado e o teste exato de Fisher^(21)^. Para amostras independentes foram utilizados o teste de Mann-Whitney, e o teste de Kruskal-Wallis. O teste de comparações múltiplas de Dunn foi aplicado após evidenciar diferenças significativas em pelo menos uma das categorias de respostas através do teste de Kruskal-Wallis.

Os resultados foram descritos e apresentados em tabelas.

## RESULTADOS

A amostra final caracterizou-se predominantemente por idosos do sexo feminino (75,1%), com faixa etária entre 66 e 75 anos (35,4%), casados (51,0%), raça/cor branca (60,3%), residindo em imóvel próprio (81,8%) localizado na zona urbana (91%), com ensino básico completo ou incompleto (31,8%), possuindo como fonte de renda aposentadoria ou pensionista (79,2%) e com um a dois dependentes de sua renda (61%) (Tabela 1).

**Tabela 1:** Características sociodemográficas em comparação ao acesso às mídias sociais. Rio de Janeiro, Rio de Janeiro, Brasil, 2022

Variáveis	Televisão				Valor de *p* ^2^	Rede Social		Valor de *p* ^2^	Rádio		Valor de *p* ^2^
	Total	SEM acesso N = 58^1^	COM acesso N = 332^1^	SEM acesso N = 231^1^		COM acesso N = 159^1^	SEM acesso N = 299^1^		COM acesso N = 91^1^		
Sexo				0,889			0,044*			0,004*
Feminino	293 (75,1)	44 (75,9)	249 (75,0)		182 (78,8)	111 (69,8)		235 (78,6)	58 (63,7)	
Masculino	97 (24,9)	14 (24,1)	83 (25,0)		49 (21,2)	48 (30,2)		64 (21,4)	33 (36,3)	
Idade				<0,001*			<0,001*			0,088
60 – 65	134 (34,4)	32 (55,2)	102 (30,7)		73 (31,6)	61 (38,4)		107 (35,8)	27 (29,7)	
66 – 75	138 (35,4)	18 (31,0)	120 (36,1)		67 (29,0)	71 (44,7)		97 (32,4)	41 (45,1)	
≥76	118 (30,3)	8 (13,8)	110 (33,1)		91 (39,4)	27 (17,0)		95 (31,8)	23 (25,3)	
Estado civil				0,093			0,017*			0,070
Casado(a)	199 (51,0)	23 (39,7)	176 (53,0)		104 (45,0)	95 (59,7)		151 (50,5)	48 (52,7)	
Viúvo(a)	73 (18,7)	10 (17,2)	63 (19,0)		53 (22,9)	20 (12,6)		54 (18,1)	19 (20,9)	
Separado(a)	67 (17,2)	16 (27,6)	51 (15,4)		43 (18,6)	24 (15,1)		59 (19,7)	8 (8,8)	
Solteiro(a)	51 (13,1)	9 (15,5)	42 (12,7)		31 (13,4)	20 (12,6)		35 (11,7)	16 (17,6)	
Coabitantes				0,176			0,299			0,192
Nenhuma	71 (18,2)	15 (25,9)	56 (16,9)		46 (19,9)	25 (15,7)		55 (18,4)	16 (17,6)	
1 pessoa	145 (37,2)	15 (25,9)	130 (39,2)		89 (38,5)	56 (35,2)		119 (39,8)	26 (28,6)	
2 pessoas	93 (23,8)	16 (27,6)	77 (23,2)		55 (23,8)	38 (23,9)		66 (22,1)	27 (29,7)	
≥3 pessoas	81 (20,8)	12 (20,7)	69 (20,8)		41 (17,7)	40 (25,2)		59 (19,7)	22 (24,2)	
Tipo de imóvel				0,009			0,478			0,353
Própria	319 (81,8)	40 (69,0)	279 (84,0)		185 (80,1)	134 (84,3)		241 (80,6)	78 (85,7)	
Alugada	46 (11,8)	14 (24,1)	32 (9,6)		31 (13,4)	15 (9,4)		36 (12,0)	10 (11,0)	
de familiar	25 (6,4)	4 (6,9)	21 (6,3)		15 (6,5)	10 (6,3)		22 (7,4)	3 (3,3)	
Nível escolaridade				<0,001|*			<0,001			0,004*
Analfabeto	9 (2,3)	0 (0,0)	9 (2,7)		8 (3,5)	1 (0,6)		4(1,3)	5 (5,5)	
Ensino básico completo	124 (31,8)	11 (19,0)	113 (34,0)		98 (42,4)	26 (16,4)		84 (28,1)	40 (44,0)	
ou incompleto										
Ensino Médio	108 (27,7)	8 (13,8)	100 (30,1)		59 (25,5)	49 (30,8)		86 (28,8)	22 (24,2)	
Graduação	70 (17,9)	14 (24,1)	56 (16,9)		27 (11,7)	43 (27,0)		58 (19,4)	12 (13,2)	
Pós-graduação	79 (20,3)	25 (43,1)	54 (16,3)		39 (16,9)	40 (25,2)		67 (22,4)	12 (13,2)	

^1^ *n (%);* ^2^ PearsonS Chi-squared test; Fisher’s exact test; * valor de p < 0,05; sd = desvio padrão.

Em relação às análises, os idosos afirmaram estarem expostos às notícias ou informações sobre COVID-19 durante a última semana frequentemente, sendo 38,5% pela televisão, 22,3% pelas redes sociais e 8,7% pelo rádio. Os meios mais citados como utilizados para acessar notícias e informações sobre COVID-19 foram: televisão (85,1%), *Whatsapp* (40,8%), sites de internet (28,7%), jornais ou revistas impressas (26,2%), *Facebook* (25,1%), rádio (23,3%), *Youtube* (14,4%), Instagram (7,7%), *Twitter* (3,3%), *Telegram* (1,8%), nenhuma das anteriores (1.5%), trabalhos científicos encaminhados (0.5%), artigos científicos (0.3%), *Google* (0.3%) e hospital de emergência (0.3%).

As variáveis sociodemográficas relacionadas com o acesso às notícias e informações sobre COVID-19, nota-se que no indicador tem afetado, na comparação de exposição entre as diferentes mídias sociais (redes sociais e televisão), elas se mostraram similares (Tabela 2). A exceção foram as variáveis idade e escolaridade que se mostraram significantes (valor de *p* <0.001). Nos grupos em que a exposição tem afetado física e/ou psicologicamente, os principais atingidos foram os idosos jovens com nível de escolaridade ensino básico e médio completos.

**Tabela 2:** Características sociodemográficas relacionadas em como o acesso às notícias e informações sobre COVID-19. Rio de Janeiro, Rio de Janeiro, Brasil, 2022

Perfil sociodemográfico	Total N = 390^1^	Redes Sociais			Valor de *p* ^2^	Televisão			Valor de *p* ^2^
		Não “utilizo” este meio de informação N = 163^1^	Não tem me afetado N = 114^1^	Tem me afetado física e/ou psicologicamente N = 113^1^		Não “utilizo” este meio de informação N = 163^1^	Não tem me afetado N = 114^1^	Tem me afetado física e/ou psicologicamente N = 113^1^		Valor de *p* ^2^
Sexo					0,347				0,640
Feminino	293 (75,1)	126 (77,3)	80 (70,2)	87 (77,0)		22 (73,3)	131 (73,)	140 (77,3)	
Masculino	97 (24,9)	37 (22,7)	34 (29,8)	26 (23,0)		8 (26,7)	48 (26,8)	41 (22,7)	
Idade					<0,001				<0,001
60 - 65 anos	134 (34,4)	31 (19,0)	40 (35,1)	63 (55,8)		12 (40,0)	42 (23,5)	80 (44,2)	
66 - 75 anos	138 (35,4)	49 (30,1)	52 (45,6)	37 (32,7)		11 (36,7)	64 (35,8)	63 (34,8)	
76 anos ou mais	118 (30,3)	83 (50,9)	22 (19,3)	13 (11,5)		7 (23,3)	73 (40,8)	38 (21,0)	
Estado civil					0,071				0,578
Casado(a)/	199 (51,0)	80 (49,1)	59 (51,8)	60 (53,1)		15 (50,0)	93 (52,0)	91 (50,3)	
morando junto(a)									
Viúvo(a)	73 (18,7)	42 (25,8)	13 (11,4)	18 (15,9)		4 (13,3)	35 (19,6)	34 (18,8)	
Separado(a)/	67 (17,2)	22 (13,5)	24 (21,1)	21 (18,6)		4 (13,3)	27 (15,1)	36 (19,9)	
desquitado(a)									
Solteiro(a)	51 (13,1)	19 (11,7)	18 (15,8)	14 (12,4)		7 (23,3)	24 (13,4)	20 (11,0)	
Coabitantes					0,177				0.280
Nenhuma	71 (18,2)	29 (17,8)	19 (16,7)	23 (20,4)		5 (16,7)	30 (16,8)	36 (19,9)	
1 pessoa	145 (37,2)	63 (38,7)	43 (37,7)	39 (34,5)		10 (33,3)	73 (40,8)	62 (34,3)	
2 pessoas	93 (23,8)	41 (25,2)	33 (28,9)	19 (16,8)		10 (33,3)	46 (25,7)	37 (20,4)	
3 pessoas ou mais	81 (20,8)	30 (18,4)	19 (16,7)	32 (28,3)		5 (16,7)	30 (16,8)	46 (25,4)	
Tipo de imóvel					0,220				0,135
Residência própria	319 (81,8)	134 (82,2)	95 (83,3)	90 (79,6)		24 (80,0)	145 (81,0)	150 (82,9)	
Residência alugada	46 (11,8)	17 (10,4)	10 (8,8)	19 (16,8)		3 (10,0)	18 (10,1)	25 (13,8)	
Residência de familiar	25 (6,4)	12 (7,4)	9 (7,9)	4 (3,5)		3 (10,0)	16 (8,9)	6 (3,3)	
Nível escolaridade					<0,001				<0,001
Analfabeto	9 (2,3)	7 (4,3)	1 (0,9)	1 (0,9)		0 (0,0)	6 (3,4)	3 (1,7)	
Ensino básico completo	124 (31,8)	79 (48,5)	23 (20,2)	22 (19,5)		0 (0,0)	66 (36,9)	58 (32,0)	
ou incompleto									
Ensino Médio	108 (27,7)	46 (28,2)	27 (23,7)	35 (31,0)		9 (30,0)	52 (29,1)	47 (26,0)	
Graduação	70 (17,9)	17 (10,4)	32 (28,1)	21 (18,6)		11 (36,7)	32 (17,9)	27 (14,9)	
Pós-graduação	79 (20,3)	14 (8,6)	31 (27,2)	34 (30,1)		10 (33,3)	23 (12,8)	46 (25,4)	

^1^ *n (%);* ^2^ Pearson’s Chi-squared test; Fisher’s exact test; * valor de p < 0,05;sd = desvio padrão.

Em relação às horas de exposição a notícias e informações sobre a COVID-19, a média de horas de exposição foi de: 5,3 horas para televisão; 6,8 horas para redes sociais (*Whatsapp, Facebook, Youtube, Instagram* e outras); e 4,7 horas para rádio (Tabela 3). Houve uma significância (p <0.001) em relação à hora de exposição e, no meio Rede Social, pontua-se que essa significância foi obtida no grupo de idosos (n = 111) que responderam ser afetados pelas notícias e informações referentes à COVID-19 veiculadas pela Rede Social. Um dado também importante para trazer à luz da discussão científica foi que o maior quantitativo de idosos (n=177) afetados pela exposição ao noticiário sobre a COVID-19 foi através do meio de comunicação Televisão.

**Tabela 3:** Horas de exposição à notícias e informações sobre COVID-19 - o tempo de exposição afeta ou não afeta o idoso. Rio de Janeiro, Rio de Janeiro, Brasil, 2022

Horas de exposição	Afeta	Psicologicamente	Fisicamente	Ambos	Valor de *p* ^2^
Rede Social	n = 111	n = 75	n = 12	n= 24	<0,001*
Média (sd)	6,8 (6,1)	7,4 (6,2)	4,4 (4,4)	6,2 (6,4)	
Mediana (q1;q3)	4,0 (2,0 – 10,0)	4,0 (3,0 – 12,0)	2,0 (1,0 – 6,2)	3,5 (2,0 – 8,0)	
Televisão	n = 177	n = 120	n = 25	n = 32	0,203
Média (sd)	5,3 (4,8)	5,1 (4,6)	4,5 (3,3)	6,6 (6,4)	
Mediana (q1;q/3)	4,0 (2,0 – 7,0)	4,0 (2,0 – 6,2)	4,0 (2,0 – 5,0)	4,5 (2,0 – 8,7)	
Rádio	n = 46	n = 33	n = 6	n = 7	0,008
Média (sd)	4,7 (5,7)	3,7 (4,3)	5,0 (2,0)	9,5 (10,5)	
Mediana (q1;q3)	3,0 (2,0 – 4,7)	2,0 (1,0 – 4,0)	5,0 (4,0 – 6,0)	3,0 (1,5 – 18,0)	

^1^ *n (%);* ^2^ Pearson’s Chi-squared test; Fisher’s exact test; * valor de p< 0,05;sd = desvio padrão.

## DISCUSSÃO

Ao mesmo tempo em que há a percepção de que esses idosos apresentam acesso aos conteúdos via internet, há uma necessidade de um maior entendimento sobre a real compreensão das informações recebidas, principalmente no período pandêmico no qual o estudo foi desenvolvido, pois é a partir dessa noção de compreensão que o impacto das diversas informações pode se tornar um fator influenciador na vida da população idosa em especial.

Outros estudos do tipo *web-based survey* conduzidos durante a pandemia no Brasil também refletem um maior número de idosos correspondentes ao sexo feminino, porém com uma faixa etária de 60 a 69 anos, casados, maior escolaridade e morando com outras pessoas. Na análise do perfil sociodemográfico da amostra do presente estudo pode-se desvelar um novo padrão entre os idosos que utilizam as mídias digitais, os quais se mostram escolarizados em um nível de compreensão e leitura que permite acessar conhecimentos via internet, corroborando com outros estudos^(22)^.

Em relação a exposição às notícias ou informações sobre COVID-19 obtida pelos idosos, a televisão foi o meio que obteve maior percentual. Dessa forma, idosos mais jovens e com maior nível de escolaridade apresentam maior tempo de exposição à televisão e também às redes sociais. Dados do IBGE mostram que os idosos que apresentam um rendimento mensal domiciliar per capita de até meio salário mínimo só alcançavam 10,8% na simultaneidade de iluminação elétrica, computador, televisão em cores e máquina de lavar^(6, 7, 8, 9, 10, 11, 12, 13, 14, 15, 16, 17, 18, 19, 20, 21, 22, 23)^.

Segundo pesquisas, para a maioria, o tempo de lazer é destinado a ver televisão, notando-se que 93% assistem televisão em casa e 72% disseram ter preferência pelas atividades desenvolvidas em casa^(24)^. Em um outro estudo desenvolvido entre idosos brasileiros, há a reafirmação do uso da televisão sobre as demais mídias. Nele, abordou-se, ainda, a preocupação em relação ao impacto da exposição demasiada sobre a saúde física, a condição psicológica e a qualidade de vida desses usuários^(25)^. Destaca-se que a televisão foi a fonte principal de informações entre as pessoas idosas moradoras das regiões Sudeste e Centro-Oeste, e a internet entre os moradores do Norte, Nordeste e Sul, assim como os achados deste estudo^(26)^.

Em relação às significâncias encontradas a partir das associações estabelecidas entre as mídias sociais e as variáveis sociodemográficas, a questão da idade foi significante em duas mídias sociais: televisão e rede social, já a escolaridade apresentou significância com a: televisão e o rádio. Analisar essas significâncias cruzadas com as variáveis sociodemográficas são fatores que influenciam no modo como os idosos compreenderam as informações recebidas e, consequentemente, a forma como tais informações poderão impactar a qualidade de saúde física e mental dos idosos.

Ao realizar uma associação das variáveis sociodemográficas, como o fator dos idosos se sentirem afetados psicologicamente, fisicamente ou ambos devido às notícias sobre COVID-19 veiculadas nas redes sociais, observa-se que a maior carga dessas alterações foi encontrada naqueles idosos com maiores níveis de escolaridade e diminuição da renda no período pós-pandemia^(27)^.

A maior parte dos idosos informou ter nível superior ou maior grau de escolaridade, algo que desperta um questionamento e um pensamento crítico ao promover a articulação do quanto os idosos podem apresentar-se afetados psicologicamente, fisicamente ou ambos, de modo que quanto mais tempo de estudo, mais impactos podem ser evidenciados diante de informações demasiadas e sem crivo científico veiculadas pelas diversas mídia.

Em uma revisão de escopo desenvolvida, foi possível identificar a associação entre maior grau de escolaridade e chance de desenvolver ansiedade e depressão. Dessa forma, inferiu-se que o grau de escolaridade poderia estar relacionado tanto à compreensão da gravidade da situação pandêmica quanto à busca por informações sobre a COVID-19^(28)^. Todavia, um outro estudo conduzido também com a população idosa da região sudeste brasileira apontou que idosos mais escolarizados apresentaram índices mais baixos de depressão e solidão devido a maiores opções estratégicas de resiliência e um suporte social^(22)^.

Há um aumento progressivo da utilização da internet entre os idosos brasileiros, chegando a 97% em 2021^(29)^. O acesso às redes sociais apresenta uma dualidade, pois, se por um lado o acesso a elas pode ocasionar maior autonomia para os idosos, mais disponibilidade de informações e fomentar as relações interpessoais, em contrapartida o mesmo acesso apresenta desafios como a infodemia, a dificuldade de controlar as informações veiculadas, fazendo assim com que o idoso não saiba qual orientação seguir podendo gerar um reflexo no impacto de sua saúde física e/ou psicológica^(30)^.

As consequências psicológicas devido ao uso excessivo das mídias sociais principalmente no período da pandemia da COVID-19 induzem a alterações cada vez mais complexas. Dentre elas, os transtornos psicológicos mais relatados nos estudos foram divididos em quatro variáveis que englobaram: ansiedade, preocupação e medo; estresse; depressão e pensamentos suicidas^(31)^. Um estudo iraquiano, realizado com 516 usuários, referente aos impactos do pânico nas mídias sociais durante a pandemia, apontou impacto significativo na saúde mental e bem-estar, principalmente na elevação dos níveis de medo, estresse, depressão e também sobre pensamentos suicidas^(32)^.

### Limitações do estudo

Uma questão limitadora do estudo foi a estratégia de coleta de dados por meio de *web-based survey,* pois mesmo muitos idosos obtendo acesso à internet, ainda há barreiras que impossibilitam os mesmos de obterem desenvoltura para efetivamente responderem uma pesquisa online. O estudo apresenta utilização de uma amostra não aleatória, se faz necessária cautela na interpretação de relações causais entre as variáveis e generalização dos achados deste estudo para a população idosa.

### Contribuições para a área da Enfermagem

É de extrema importância que ocorra a investigação das questões referentes às mídias sociais e o quanto elas podem afetar, impactar a saúde dos idosos em especial nos períodos de pandemia como foi a pandemia de COVID-19. O estudo faz emergir como contribuição a necessidade constante de discussão de evidências científicas referentes a questões que alteram a saúde mental e física da população idosa, para, assim, contribuir com as políticas públicas voltadas para promoção e manutenção do bem estar psicológico e físico dos idosos. Além disso, a pesquisa propõe novos estudos com estratégias de minimização dos impactos negativos que possam surgir com a infodemia de COVID-19.

## CONCLUSÕES

As alterações físicas e psicológicas existem efetivamente e estão correlacionadas com a exposição às informações sobre a COVID-19 na vida dos idosos, o que possibilitou a identificação do impacto sofrido por esta população. Embora seja uma temática contemporânea, ainda há necessidade de uma maior exploração multiprofissional e em especial pela enfermagem gerontológica, tendo em vista que qualquer tipo de alterações físicas e psicológicas vão interferir no perfil de saúde dos idosos. O entendimento dessas repercussões é necessário para que se proponha intervenções efetivas no âmbito individual e coletivo para o enfrentamento da infodemia nesse grupo populacional.

É de primordial necessidade que sempre possa haver discussões científicas acerca da saúde mental e bem-estar da população, principalmente em momentos tão específicos como a pandemia de COVID-19. Assim, o estudo possibilitou visualizar as alterações físicas e psicológicas da infodemia de COVID-19 para a população idosa do Rio de Janeiro e ainda pôde validar a significância das mídias sociais para os idosos e o quanto as notícias veiculadas afetam esta população.
